# Prescribing equity: physicians as advocates for access to essential medicines. A call to action from medical graduates

**DOI:** 10.1177/20499361241281136

**Published:** 2024-10-18

**Authors:** Emmanuel Adams-Gelinas, Amanda Bianco, Virginie Boisvert-Plante, Santina Conte, Inès Dupuis, Anda Gaita, Lina Hadidi, Yutong Huang, Meryem Jabrane, Kimiya Kaffash, Loubna Lamrani, Marine Leblanc, Sara Marier, Alexander Moise, Chloe Pereira-Kelton, Amélie Rochon, Shanti Rumjahn-Gryte, Veronika Svistkova, Marie-Catherine Viau, Kiana Yau

**Affiliations:** Faculty of Medicine and Health Sciences, McGill University, Montreal, QC, Canada; Faculty of Medicine and Health Sciences, McGill University, Montreal, QC, Canada; Faculty of Medicine and Health Sciences, McGill University, Montreal, QC, Canada; Faculty of Medicine and Health Sciences, McGill University, Montreal, QC, Canada; Faculty of Medicine and Health Sciences, McGill University, Montreal, QC, Canada; Faculty of Medicine and Health Sciences, McGill University, Montreal, QC, Canada; Faculty of Medicine and Health Sciences, McGill University, Montreal, QC, Canada; Faculty of Medicine and Health Sciences, McGill University, Montreal, QC, Canada; Faculty of Medicine and Health Sciences, McGill University, Montreal, QC, Canada; Faculty of Medicine and Health Sciences, McGill University, Montreal, QC, Canada; Faculty of Medicine and Health Sciences, McGill University, Montreal, QC, Canada; Faculty of Medicine and Health Sciences, McGill University, Montreal, QC, Canada; Faculty of Medicine and Health Sciences, McGill University, Montreal, QC, Canada; Faculty of Medicine and Health Sciences, McGill University, Montreal, QC, Canada; Faculty of Medicine and Health Sciences, McGill University, Montreal, QC, Canada; Faculty of Medicine and Health Sciences, McGill University, Montreal, QC, Canada; Faculty of Medicine and Health Sciences, McGill University, Montreal, QC, Canada; Faculty of Medicine and Health Sciences, McGill University, Montreal, QC, Canada; Faculty of Medicine and Health Sciences, McGill University, Montreal, QC, Canada; Faculty of Medicine and Health Sciences, McGill University, Montreal, QC, Canada

**Keywords:** access to medicine, equity, intellectual property, physician advocacy, vaccine apartheid

## Correspondence

From gene therapies for sickle cell disease to mRNA vaccines for COVID-19, medical innovation has few boundaries.^1,2^ Yet, billions of people suffer or die because the vaccines, medicines, and diagnostic tests that they need are either unavailable or unaffordable.^3^ Why is access to essential medical products so restricted? Why do the fruits of scientific innovation fail to reach such a big proportion of our world’s population? This systemic failure poses a particularly concerning threat to humanity in our day and age, where the climate and biodiversity crises collide with incessant violent conflicts resulting in an exponential increase of the risk of future pandemics.

In the present context, greed seems to prevail over the health needs of the world, as demonstrated by the intellectual property that primarily benefits rich nations and corporations. Policymakers in high-income countries resist the loosening of patent regulations, even during global emergencies, with the false excuse that it protects innovation.^4,5^ They choose to enable drug market monopolies that render medications unaffordable and to blunt epidemic countermeasures by allowing the gatekeeping of research and development. All of this favors the profit of pharmaceutical companies, thereby perpetuating inequities around the globe, and exacerbating existing hardship, especially in low-income and middle-income countries (LMICs). Governing bodies could instead promote collective intelligence and ownership, knowledge sharing, and as needed, technology transfer among nations to favor health equity around the globe—in the way of a common good approach.^6,7^ Unfortunately, Big Pharma’s lobbying has more influence on governments than the health of the public. As a result, patients and physicians alike are left discouraged. The recent COVID-19 pandemic provides a morbid example of the struggles to access essential medical products, still rampant to this day. High-income countries created a “vaccine apartheid” by hoarding 870 million excess doses while entire continents—namely Africa—were prevented from reaping their share of the rewards of health innovation, resulting in millions of preventable deaths.^8^ We are far from the principles of serving the common good, while structural inequities persist and prevail, in the name of profit.

Fortunately, physicians can play a key role in reversing the tide. The first step in improving access to medications is to recognize that cost may pose a barrier for certain patients. Healthcare institutions manage individuals from diverse socioeconomic statuses. Physicians should be mindful of each patient’s financial situation and insurance coverage when prescribing a new medication to ensure the feasibility of compliance with the treatment of each patient. It is imperative for doctors to remain up to date on the latest developments in medications and vaccines to make informed prescribing practices, such as opting for quality generic drugs when available and affordable. Too often, physicians engage in misinformed prescribing practices when they rely on drug companies to teach them about new drugs and their potential benefits. Even if physicians and the pharmaceutical industry share the common goal of advancing medical knowledge, profitability remains the pharmaceutical companies’ main objective. Pharmaceutical companies can meddle with the clinical decision-making of physicians by offering financial compensation and, in doing so, jeopardize the quality of patient care, the affordability of healthcare as well as the patient–physician relationship. A recent scoping review by Frontiers in Public Health described that pharma’s payments to physicians lead to an increased prescription of low-value, expensive, and brand-name drugs as well as rapid prescription of new drugs—ultimately leading to an overall increase of drug prices.^9^ This highlights a conflict of interest with drug companies promoting their products—and influencing physicians—even if they are not the best or cheapest option for patients.

However, the duty of physicians extends past their clinic or hospital. Physicians need to collaborate with policymakers, pharmaceutical companies, and advocacy groups to address systemic barriers to medication access. The adoption of the Trade-Related Aspects of Intellectual Property Rights (TRIPS) agreement in 1995, which set global standards on intellectual property rights, inhibited the generic medicine industry from offering new drugs at lower prices.^10^ This World Trade Organization (WTO) accord was negotiated without any input from the healthcare community, especially communities in the Global South.^11^ Around the same time, the HIV epidemic was on the rise globally and patented-protected, life-saving antiretrovirals (ARVs) had just become available at exorbitant prices of US$ 10,000–15,000 per patient per year. As a response, many activists and health professionals advocated for increased flexibility in the application of patent laws to protect public health and promote access to essential and life-saving medicines.^12^ Over the next few years, multiple generic companies, particularly in India, started making ARVs and contributed to temporarily dropping the costs related to these medications in LMICs. This was possible until the Patent Act of TRIPS was amended in India in 2005 to include product patents in addition to the previously recognized process patents, hence inhibiting the manufacturing of generic products by reverse-engineering.^13^ Healthcare professionals’ crucial role in the world uniting to make ARVs more accessible highlights the importance of physician advocacy in making essential medicines affordable, but this historical success merely addresses one facet of a huge societal issue.

Physicians are first-line witnesses of the struggles that some patients face to afford essential and life-saving treatments. In addition, many doctors are experts in public health and infectious diseases. Hence, they are in a unique position to demand better access to medication by talking to their political representatives. Physicians need to plead with members of government and policymakers, requesting that official legislation be put in place to limit the profit margin that can be drawn on essential medications, especially if the pharma companies benefit from public funding. How ridiculous is it that the Chief Executive Officer of Moderna made $398 million in 2022 while billions of people worldwide, the majority from LMICs, were struggling to access the COVID-19 vaccines produced by his company?^14^ Such a blatant contrast highlights the need to plead for transparency in pricing and policies for price-cap regulations—such as by requesting the sharing of the net prices of health products and reporting of sales revenues, prices, units sold, marketing costs, and more; as advocated for in the World Health Organization resolution of 2019.^15^ Physicians can bring their expertise and credibility to grassroots advocacy groups, thereby amplifying their impact on the decrease in drug prices worldwide, as illustrated by the response to the HIV epidemic.

As discussed earlier, the COVID-19 pandemic exposed the ongoing inequity behind the development, supply, and distribution of vaccines worldwide—serving as a sad example of discriminatory intellectual property legislation. In response to the asymmetric international access to COVID-19 vaccines in 2020, a temporary suspension of the TRIPS agreement was proposed at the WTO.^16^ This waiver would have allowed LMICs, who did not have the purchasing power to secure vaccines from the oligopoly, to access the technologies necessary to internally produce and distribute diagnostic tests and vaccines. While this waiver was supported by many physician associations globally, including Doctors Without Borders, many rich countries unfortunately opposed the waiver.^17^ As a result, negotiations were delayed and only a limited—and disappointing—version of the waiver was agreed on by WTO members. While the leadership of big pharmaceutical companies was seeing unimaginable profits, LMICs were forced to waste valuable time in the fight against the virus in their attempts to reverse engineer the privatized formula of the COVID-19 vaccines, despite its development being heavily publicly funded.^18^ Yet again, the greed of a few international players hindered an equitable and timely public health response to a major health crisis—and cost millions of precious lives.^8^

The inequities in accessing appropriate treatments and diagnostic tests, deeply rooted in the capitalistic pharma model, are not limited to LMICs. Even in rich nations, such as the United States, access to essential medical products proves to be a massive challenge, especially for patients from lower socioeconomic backgrounds. Essential medications are sometimes inaccessible even in Canada with one in four diabetic Canadians being unable to follow their treatment plan due to cost.^19^ Despite a renowned universal health coverage, it is only now, in 2024, that the Canadian government is announcing the National Universal Pharmacare plan.^19^ As a starting point, the federal government is committed to fully covering some diabetic treatments and contraception, rendering these medications free for people across the country. This is the first step in tearing down the cost barrier that surrounds medications to ensure their accessibility for all, irrespective of economic status and geographical location in Canada. Physicians need to voice their support for such initiatives and advocate for further implementation of coverage for additional medications under this regime. Along the same lines, they should demand policies that empower them to participate in political decisions impacting healthcare. Creating dedicated positions for healthcare professionals in decision-making bodies can ensure that equity and social justice considerations are prioritized in pharma care policymaking.

Ultimately, inequities of access to affordable medical products cannot be truly solved through trade agreements, policies, and considerate prescriptions only. For radical change to happen, physicians and activists need to adopt different angles, which includes mobilizing from within pharmaceutical companies themselves. It is important to remember that pharmaceutical companies need physicians as much as physicians need pharmaceutical companies. Physicians should attempt to force change from within the pharma industry and address systemic barriers such as elevated costs, patent monopolies, and limited insurance coverage by occupying leadership positions within pharma, whether in medical affairs, market access, or clinical R&D. The fixation on money and power must be confronted from within and replaced by an all-around devotion to justice and health.

We can imagine an ideal where physicians prioritize prescribing the most effective and affordable medications to their patients, start advocating for dedicated health worker positions in decision-making bodies, and start occupying leadership positions within pharmaceutical companies. For this scheme to be beneficial and realistic, a reinforcement of equity principles within the general healthcare and academia ecosystem would also be required. Indeed, for physicians to become good instigators of change, it first requires a revisited thoughtful training curriculum and a strong social justice culture within health institutions and academia. It would also require a general wariness of Big Pharma from all physicians when consulting the health literature, gathering scientific information, and receiving updates on the latest medical treatments. Compounding on the above cultural and institutional changes, then we can imagine physicians directly influencing the problematic structures distorted by greed and hopefully promoting alternative models of R&D and better drug pricing.

The most crucial lesson to remember is that having access to essential and life-saving medicines represents a fundamental human right. Every individual on this earth deserves to live a healthy life. Patients’ access to medicine should be solely dictated by their health needs rather than their socioeconomic status, ethnicity, or any other discriminatory factor. This principle promotes individual well-being and strengthens public health outcomes. In return, this fosters economic productivity and contributes to overall societal resilience. Dismantling the systemic barriers to accessing essential medical products and cultivating equitable healthcare systems worldwide should therefore be a major priority for health professionals. Physicians must call out the inhumane act of gatekeeping essential and life-saving medicines, advocate for global health equity, and take action to change the frameworks that allow for these inequities to be perpetuated. Only then can we start imagining a world where people are prioritized over profits.

## Declarations

**Ethics approval and consent to participate:** Not applicable.

**Consent for publication:** Not applicable.

**Availability of data and materials:** Not applicable.
